# Endoscopic submucosal dissection of a laterally spreading tumor totally invading a deep sigmoidal diverticulum using an adaptive traction strategy

**DOI:** 10.1055/a-2114-0763

**Published:** 2023-07-13

**Authors:** Jean Grimaldi, Bertrand Napoléon, Louis-Jean Masgnaux, Sarah Leblanc, Jérôme Rivory, Vincent Lépilliez, Mathieu Pioche

**Affiliations:** 1 Gastroenterology and Endoscopy Unit, Fleyriat Hospital, Bourg-en-Bresse, France; 2 Gastroenterology and Endoscopy Unit, Jean Mermoz Hospital, Lyon, France; 3 LabTAU Inserm U1032, Lyon, France

Endoscopic resection of colonic lesions invading diverticula is technically challenging due to the absence of an underlying muscle layer. It has been shown that the endoscopic mucosal resection (EMR) technique is associated with significant risks of perforation and failure for diverticular lesions 1 . The endoscopic submucosal dissection (ESD) technique with traction strategy seems to be associated with more promising results for shallow diverticula (< 6 mm) 2 . However, for deeper diverticula, incomplete resection rates are high owing to the technical difficulty, and only few previous successes have been described for these lesions 3 .

We report here the case of a 73-year-old patient referred for resection of a 4.5-cm granular laterally spreading tumor (LST) ( Fig. 1 ) totally invading a deep sigmoidal diverticulum (type 3, > 1 cm). An ESD was performed using the ATRACT 2 + 2 adaptive multi-traction device ( Fig. 2 ) 4 5 . After a circumferential incision was made and the dissection started ( Fig. 3 ), the force of traction was increased by tightening the device to evaginate the diverticulum within the colonic lumen ( Fig. 4 ) in order to dissect the entire diverticulum ( Video 1 ).

**Fig. 1:**
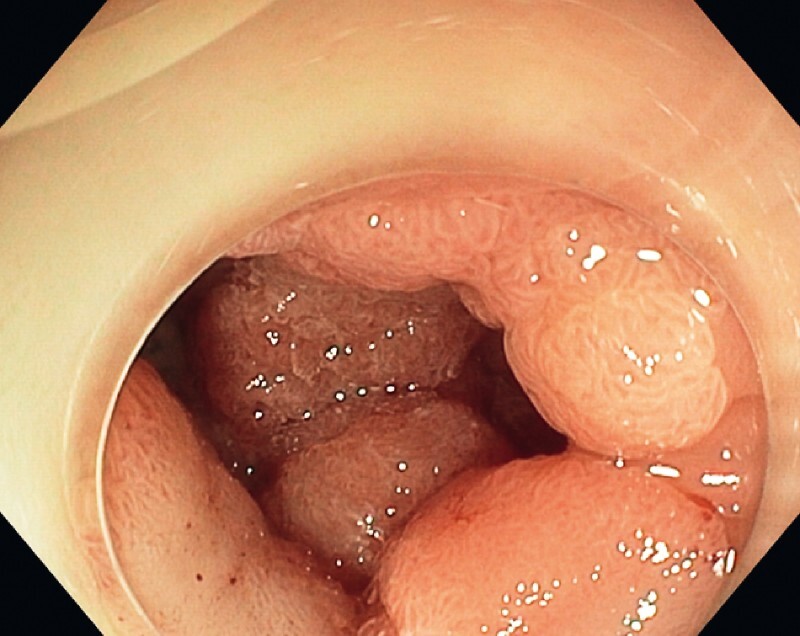
A 4.5-cm granular laterally spreading tumor totally invading a deep sigmoidal diverticulum.

**Fig. 2:**
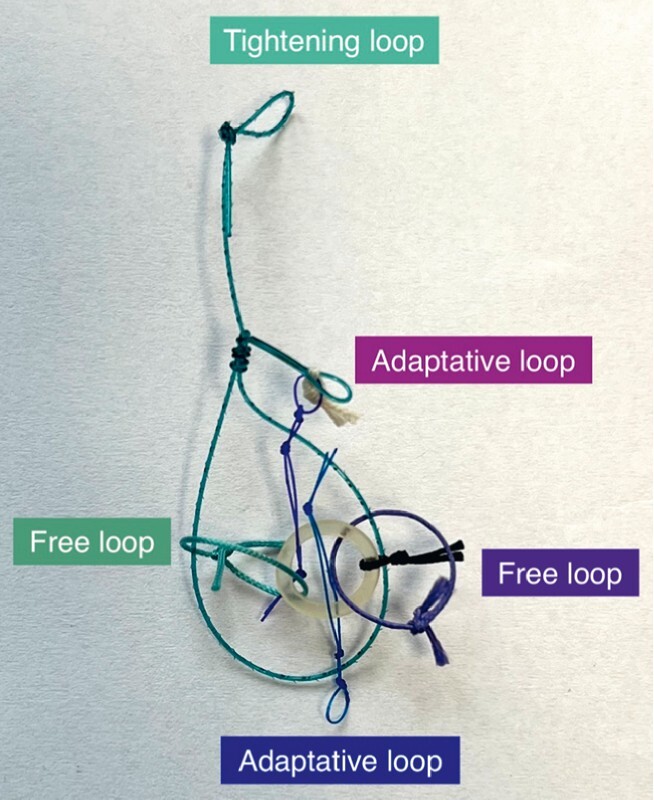
A-TRACT 2 + 2 device.

**Fig. 3:**
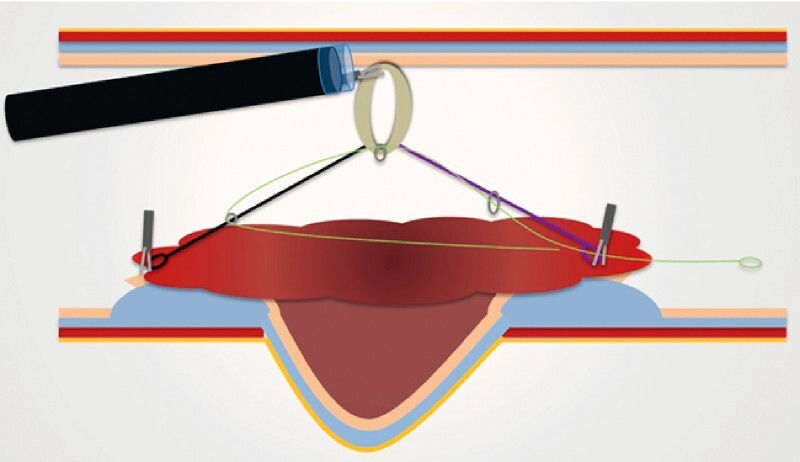
First phase of the resection strategy: placement of the traction device on the edges of the lesion to begin dissection.

**Fig. 4:**
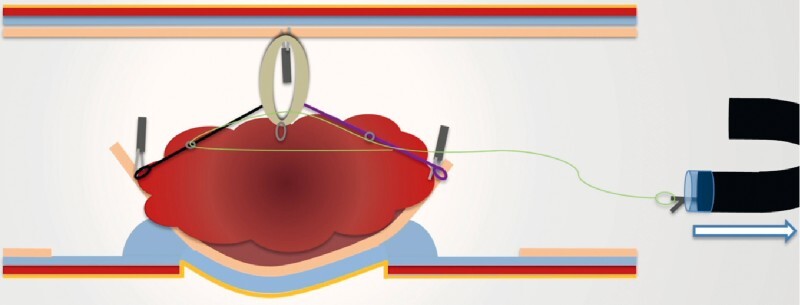
Second phase of the resection strategy: once the extra-diverticular part of the lesion has been dissected, tightening of the device to evaginate the diverticulum within the colonic lumen.

**Video 1** Endoscopic submucosal dissection of a laterally spreading tumor totally invading a deep sigmoid diverticulum using an adaptive traction strategy.


The procedure allowed a total resection of the lesion with healthy margins. It corresponded to a low grade dysplasia adenoma. There were no complications during the procedure, including no perforation.

Further investigations are needed to evaluate the efficacy of the adaptive traction strategy for the resection of diverticular colonic tumors, but the technique seems promising, especially for deep diverticula.

The adaptive traction technique appears to hold great promise in difficult-to-localize lesions such as diverticular lesions. It allows increasing the intensity of the traction throughout the resection, and in particular to evaginate deep diverticula thanks to maximal traction power at the end of the procedure. Further investigations will be necessary to evaluate the effectiveness of this strategy on a series of patients.

Endoscopy_UCTN_Code_TTT_1AQ_2AD

## Notes

Endoscopy E-Videos

https://eref.thieme.de/e-videos
*E-Videos* is an open access online section of the journal *Endoscopy* , reporting on interesting cases and new techniques in gastroenterological endoscopy. All papers include a high-quality video and are published with a Creative Commons CC-BY license. Endoscopy E-Videos qualify for HINARI discounts and waivers and eligibility is automatically checked during the submission process. We grant 100% waivers to articles whose corresponding authors are based in Group A countries and 50% waivers to those who are based in Group B countries as classified by Research4Life (see: https://www.research4life.org/access/eligibility/ ). This section has its own submission website at https://mc.manuscriptcentral.com/e-videos
